# Development of a Sandwich ELISA for EHEC O157:H7 Intimin γ1

**DOI:** 10.1371/journal.pone.0162274

**Published:** 2016-09-07

**Authors:** Xuehan Zhang, Meng Li, Bicheng Zhang, Kangming Chen, Kongwang He

**Affiliations:** 1 Institute of Veterinary Medicine, Jiangsu Academy of Agricultural Sciences, Key Laboratory of Engineering Research of Veterinary Bio-products of Agricultural Ministry, National Center for Engineering Research of Veterinary Bio-products, Nanjing 210014, China, Jiangsu Co-innovation Center for Prevention and Control of Important Animal Infectious Diseases and Zoonoses, Yangzhou, 225009, China, Key Lab of Food Quality and Safety of Jiangsu Province-State Key Laboratory Breeding Base, Nanjing 210014, China; 2 Department of Diagnostic Medicine/Pathobiology, Kansas State University, Manhattan, KS 66506, United States of America; Agricultural University of Athens, GREECE

## Abstract

Enterohemorrhagic *Escherichia coli* (EHEC) O157:H7 is a zoonotic pathogen of worldwide importance that causes foodborne infections in humans. Intimin gamma 1 (intimin γ1) is one of the most important outer membrane proteins required for EHEC’s intimate adhesion to epithelial cells. Here, we generated a polyclonal antibody (pAb) and a monoclonal antibody (mAb) against intimin γ1 to develop a double antibody sandwich ELISA (DAS-ELISA) with increased sensitivity and specificity for measuring EHEC O157:H7. To achieve this goal, a rabbit pAb was used as a capture antibody, and a mouse mAb was a detection antibody. No cross-reactivity was observed with the other genera of pathogenic bacteria tested with the DAS-ELISA, which included *Salmonella enteritidis*, *Shigella flexneri* type 2, *Listeria monocytogenes*, *Streptococcus suis* type 2, and other 18 serotype *E*. *coli*. Detection limits of the DAS-ELISA were 1 × 10^3^ CFU/mL for EHEC O157:H7 cultures, 1 × 10^4^ CFU/g before enrichment, and 1 × 10^2^ CFU/g after enrichment of contaminated samples. Field samples (n = 498) were tested using a previously established duplex-PCR method and compared to our DAS-ELISA. The DAS-ELISA had a specificity of 94.4%, a sensitivity of 91.5% and accuracy of 94.0% compared with duplex-PCR. The DAS-ELISA developed here can be applied to EHEC O157:H7 quantification in food, animal, and environmental samples.

## Introduction

Enterohemorrhagic *Escherichia coli* (EHEC) O157:H7 is a zoonotic pathogen of worldwide importance that causes foodborne infections in humans [1]. Ruminants, which are asymptomatic carriers, are considered to be a major source of EHEC O157:H7, which is spread through fecal contamination of food (i.e. animal meat, milk, and vegetables) [2].

Intimin is encoded by the *E*. *coli* attaching and effacing (*eae*) gene, which is required for intimate adhesion to epithelial cells and cytoskeletal reorganization. The N-terminal region of the Intimin molecule is conserved, and the variable C-terminal sequence of Intimin defines at least 27 different Intimin subtypes [3–5]. EHEC O157:H7 is characterized by the Intimin γ1 subtype and therefore its C-terminal fragment is used as a target to detect EHEC O157:H7.

To elucidate the epidemiology of EHEC O157:H7 infections, many methods have been developed. The selectivity of sorbitol MacConkey agar (SMAC) and enrichment broths are based on specific phenotypic characteristics of most EHEC O157:H7 strains, but for some strains lacking sorbitol fermentation, failure to produce β-glucuronidase causes false negatives. Current nucleotide detection methods are based on amplifications of specific virulent genes from EHEC O157:H7 or conserved genes from EHEC strains. Genetic markers used for PCR and real-time assays to measure EHEC O157:H7 include Shiga toxin (*stx*1 and *stx*2) [6, 7], *E*. *coli* β-glucuronidase (*uid*A) [8, 9], *eae* [10, 11], fimbriae A (*fim*A) [12], Z3276 (a conserved ORF in EHEC O157:H7) [13]**,** O antigen transporter (*rfb*E), and H antigen (*fli*C) [14, 15]. However, target genes were amplified from dead cells and included contaminated nucleotides from other EHEC strains, causing overestimation of EHEC O157:H7 carriers in clinical samples. Immunology-based assays have been developed for measuring specific antigens generally expressed on the outer membrane and secreted proteins from EHEC O157:H7. Kerr’s group (2001) developed a sandwich ELISA based on long-chain lipopolysaccharide to assay EHEC O157 from clinical samples, but cross reactivity with other bacteria occurred [16]. Here, we produced a polyclonal (pAb) and monoclonal antibody (mAb) against Intimin γ1 for the purpose of developing a sandwich ELISA to measure EHEC O157:H7 in clinical samples.

## Materials and Methods

### Samples and animals

Vegetables (e.g. lettuce, spinach, sprouts, and water chestnuts), 90% lean ground beef, and fish (blackfish and bass) were purchased from three Xiaolingwei Market in the eastern part of Nanjing, the Zhongcaiwuliu wholesale food market in southern Nanjing, and the Liuhe vegetable market in northern Nanjing, and they were placed in a plastic zipper bag and kept on ice.

Raw milk and feces from dairy cattle were collected from farms with documented bio-security measures. Dairy cattle were healthy with no diarrhea or mastitis and aged 1 year to 3 years of age from 3 local dairy farms (Tangshan, Xigang, and Tianchang Farms) were sources of raw milk and feces. Cattle were fed a diet containing ~25% grain, 12% soybean meal, 3% supplements, 40% silage, and 20% alfalfa hay. Fresh feces were collected with sterile gloves, and samples were placed in plastic tubes with 10% glycerol PBS. Raw milk was collected by milking each animal with sterile gloves and placing the contents in a 2-liter flask which was kept on ice during transportation.

Pig feces were collected from farms documented to be applying good bio-security measures. Healthy pigs were from a Luhe farm in Nanjing were on a standard diet produced by Dabeinong Company. Fresh excretive feces were collected using sterile gloves and placed in plastic tubes with 10% glycerol PBS.

### Bacterial strains

The bacteria used in this study are listed in Table 1 and were tested using a double antibody sandwich ELISA (DAS-ELISA) to measure specificity. EHEC O157:H7 EDL933, isolated from ground beef linked to toxic hamburger meat in Michigan in 1982, was a gift from Dr. Dai Jianjun, Nanjing Agricultural University, and contained well-characterized intimin γ1.

**Table 1:** Bacteria used in this study.

Number	Strain/serotype	Origin	*eae* types
1	*Salmonella enteritidis*	CVMCC*	N*
2	*Shigella flexneri* type 2	CVMCC	N
3	*Listeria monocytogenes*	CVMCC	N
4	*Streptococcus suis* type 2	pig isolate	N
5	EHEC O157:H7 XG	cattle isolate	γ1
6	EHEC O157:H7 JSC1	cattle isolate	γ1
7	EHEC O157:H7 CWN11	water chestnut isolate	γ1
8	*E*. *coli* O157:H45	cattle isolate	α
9	*E*. *coli* O26:H11	fish isolate	β
10	*E*. *coli* O128:H2	poultry isolate	β
11	*E*. *coli* O111:H-	cattle isolate	γ2
12	*E*. *coli* O103:H2	cattle isolate	ε
13	*E*. *coli* O113:H-	water isolate	η
14	*E*. *coli* O145:H4	water isolate	ι
15	*E*. *coli* F4	CVMCC	N
16	*E*. *coli* F5	CVMCC	N
17	*E*. *coli* F6	CVMCC	N
18	*E*. *coli* F41	CVMCC	N
19	*E*. *coli* O138	CVMCC	N
20	*E*. *coli* O139	CVMCC	N
21	*E*. *coli* O141	CVMCC	N
22	*E*. *coli* BL21(DE3)	Takara	N
23	*E*. *coli* DH5α	Takara	N
24	EHEC O157:H7 EDL933	gift	γ1
25	BL21(DE3)/pCold I -C1*eae*	This study	N
26	BL21(DE3) /pCold I -C2*eae*	This study	N

*N, represents no *eae* genes;

*CVMCC, China Veterinary Microbiological Culture Collection center.

All bacteria used were sub-cultured twice before use.

### Antigen cloning, expression and purification

The PCR product of the 900 bp (1903–2802 nt) and 360 bp (2443–2802 nt) C-terminal sequence of *eae*-γ1 were amplified from chromosomal EHEC O157:H7 EDL933 and introduced into pCold I to generate recombinant bacteria BL21(DE3)/pCold I-C1*eae* and BL21(DE3)/pCold I-C2 *eae*, and they express recombinant proteins C1-intimin γ1 and C2-intimin γ1. Bacterial cultures were grown overnight at 37°C and subcultured (1:100) into fresh media. Subcultured cells were grown for 2 h at 37°C until reaching an optical density (OD_600_) of 0.7–0.8, at which time isopropyl β-d-thiogalactopyranoside (IPTG) (0.5 mM) was added and incubation was continued for 6 h. After bacterial cultures were harvested by centrifugation and resuspended in PBS, containing 1 mM Pefabloc, 0.5 mg/mL lysozyme, 10 μg/mL DNase I, and 10 μg/mL RNase A. Cell lysates were ultrasonicated for 5 min with 30 s intervals on ice. Centrifuged supernatants were purified using His•Bind Resin Chromatography according to the manufacturer’s instructions (Macherey-Nagel Corp., Germany).

### Preparation of pAb against C1-Intimin γ1

Laboratory animal experimentation was performed in compliance with the Jiangsu Administration Guidelines for the Use of Experimental Animals. All procedures were approved by the Animal Ethics Committee of Jiangsu Institute of Veterinary Medicine (SYXK20120301). New Zealand white rabbits were obtained from Jinling rabbit Farm (Nanjing, China), housed in cage (W 50 cm, L30 cm, H40 cm). They were provided with food and water *ad libitum*. Throughout the course of this study, no rabbits were ill and died prior to experimental endpoint. At the end of the study, they were euthanized by intravenous injection of air, exsanguinated, and blood was clotted at 20°C for 2 h, chilled at 4°C for overnight. The pAb against C1-Intimin-γ1 was produced by subcutaneously injecting the rabbits. First, the C1-intimin-γ1 was emulsified with either complete Freund’s adjuvant (1st immunization) or incomplete adjuvant (2nd and 3rd boosters) prior to immunization. The emulsion was injected into the rabbit back at 3-week intervals (500 μg protein each). After the 3rd injection, blood was collected to measure antibody titers using indirect-ELISA [17]. ELISAs were performed in 96-well plates (Costar, USA) coated with C1-Intimin-γ1 antigen (2.5 μg/mL) and incubated at 4°C overnight. Plates were washed and blocked with 1% bovine serum albumin (BSA) in phosphate buffered saline containing 0.05% Tween 20 (PBST). 100 μL of serially diluted rabbit negative sera and antisera was added to antigen-coated wells and incubated at 37°C for 1 h. Sera were removed prior to adding goat anti-rabbit IgG-HRP (1/5000 in PBST) (Boster, Wuhan, China) for 45 min at 37°C. Plates were washed and substrate solution TMBS (Sigma) was added for 5 minutes at 37°C followed by 2 M H_2_SO_4_. Absorbance was read at 450 nm. Antibody titer was defined as the reciprocal of the highest dilution of serum producing 2:1 ratio value above negative levels. Antisera raised against C1-Intimin-γ1 antigen were purified using a Protein A IgG Purification Kit (ThermoFisher) and stored in -80°C freezer. Quick Start^™^ Bradford Protein Assay (Bio-Rad) kit was used to measure pAb concentration according to manufacturer’s instructions.

### Preparation of mAb against C2-Intimin γ1

Laboratory animal experimentation was performed in compliance with the Jiangsu Administration Guidelines for the Use of Experimental Animals. All procedures were approved by the Animal Ethics Committee of Jiangsu Institute of Veterinary Medicine (SYXK20131002). BALB/c mice were bought from experimental animal center of Yangzhou University (Yangzhou, China), housed in microisolator cage, provided with food and water *ad libitum*. During feeding and study, health status of mice was monitored twice a day and recorded the clinical signs (ruffled hair coat, hunched posture, inflamed injection site). If animals displayed clinical signs of illness, they were euthanized by cervical dislocation. Mice were immunized by injecting 50 μg (i.p.) purified C2-Intimin γ1 mixed with the same volume of ISA50V adjuvant (Seppic Corp.) on three occasions at 3-week intervals. The final injection was given with 30 μg protein, and mice were killed 3 days later and spleens and lymphocytes were fused with SP2/0 myeloma cells to generate hybridomas. The procedures of cell fusion, hybridomas screening and cloning conditions were described previously by Ko¨hler. et al. [18] and Hao chen, et al. [19]. Hybridomas significantly reactive to C2-Intimin γ1 were selected for sub-cloning. Ascites fluid containing antibodies was produced from cloned hybridoma lines by injecting liquid paraffin and 5×10^5^ cells into BALB/c mice. After 10 days, ascites were harvested, purified using Protein A IgG Purification Kit (ThermoFisher) and stored in -80°C freezer. Quick Start^™^ Bradford Protein Assay (Bio-Rad) kit was used to measure mAb concentration according to manufacturer’s instructions.

### Development of DAS-ELISA

A checkerboard test was used to identify the optimal concentration of capture and detection antibody. Rabbit pAb against C1-intimin-γ1 IgG was the capture antibody and mouse mAb against C2-intimin γ1 IgG was the detection antibody. Briefly, plates were coated with pAb and mAb at 20, 10, 5, 2.5, 1.25, and 0.625 μg/mL to identify the optimal combinations. EHEC O157:H7 EDL933 strain cultures (10^6^ CFU/mL) and PBS were used as positive and negative antigens, respectively. DAS-ELISA was performed by the following procedure. Briefly, a 96-well microtiter plate (Costar, USA) was coated with 100 μL/well of the capture antibody in coating buffer (0.01 M sodium carbonate buffer, pH 9.6 and 0.01 M PBS, pH 7.4) at 4°C overnight. The plate was washed with 0.01 M PBST and added with 250 μL of blocking buffer (5% skim milk, 1% gelatin, and 1% BSA) at 37°C for 2 h. After washing with PBST to remove blocking buffer, 10^6^ CFU/mL of EHEC O157:H7 EDL933 strain cultures were added and incubated at 37°C for a couple of hours (1 h and 2 h). Wells were washed and detection antibody diluted in PBST was added, followed by incubation at 37°C for 1 h. The plate was washed as above and 100 μL of HRP-conjugated goat anti-mouse IgG diluted 1:10,000 in PBST was added and incubated at 37°C for 1 h. After the final wash, 100 μL substrate TMBS was added to promote an enzyme-substrate reaction at 37°C for 10 min. The reaction was stopped with 50 μL stop solution of 2 M H_2_SO_4_. Absorbance was read at 450 nm with an ELISA plate reader. The optimal condition was determined by comparing the positive/negative ratio (P/N) of the samples.

### Sensitivity and Specificity test

DAS-ELISA sensitivity was evaluated with two types of EHEC O157:H7 samples. Serial dilutions of 10^9^ CFU/mL to 10^2^ CFU/mL of EHEC O157:H7 EDL933 strain cultures were used for this assay. The artificially contaminated samples were detected by the DAS-ELISA and duplex-PCR as previously described [20]. Artificially contaminated samples were prepared by following procedure. In brief, ground beef and fecal samples were divided into 25-g portions and placed into sterile flasks, respectively. Lettuce was cut into pieces and separated into 25-g portions and placed in flasks. Lettuce, ground beef, and fecal samples were examined for preexisting EHEC O157:H7 contamination using the DAS-ELISA and duplex-PCR plus a modified EC broth (mEC, Sigma-Aldrich) containing 100 μg/mL novobiocin and 1% tellurite [21]. Pathogen-free samples were then used to prepare artificially contaminated samples. Two hundred and fifty microliters of the EHEC O157:H7 EDL933 strain cultures of 1×10^11^ CFU/mL were added to flasks containing 25 g samples and 50 mL of PBS, and samples were mixed well to achieve target inoculum level of 10^9^ CFU/g followed by serial dilution of 10^8^ CFU/g, 10^7^ CFU/g, 10^6^ CFU/g, 10^5^ CFU/g, 10^4^ CFU/g, 10^3^ CFU/g, and 10^2^ CFU/g. Specimens were homogenized for 30 min with shaking and 100 μL of samples was used for the DAS-ELISA assay based on the results. Negative samples were enriched with mEC broth containing novobiocin and tellurite at 41°C for 12 h and centrifuged to remove particles. After aspirated supernatants were centrifuged at 12,000 × g for 10 min, pellets were suspended and diluted with 0.2 mL TE buffer. Finally, 100 μL of the suspended pellets was used to repeat detection by DAS-ELISA and 100 μL were used to extract a template for analysis using duplex-PCR as previously described [20].

To evaluate the specificity of the DAS-ELISA, pure cultures of *Salmonella enteritidis*, *Shigella flexneri* type 2, *Listeria monocytogenes*, *Streptococcus suis* type 2, and 20 *E*. *coli* isolates representing 17 serotypes were tested using the DAS-ELISA. For parallel comparison with these strains, 10^6^ CFU/mL bacterial cultures were used to prepare samples. The specificity of the method was evaluated based on the results.

### Threshold determination

Fifty negative fecal samples from uninfected calves were detected by established DAS-ELISA using optimal conditions described in the results. Measurements were done in duplicate on different plates and the mean value of them was taken as the readout. The DAS-ELISA assay cut-off value at OD_450_ was calculated from all negative samples as the mean value plus three standard deviations (SD): mean + 3 SD. Samples with 450 nm values equal to or greater than a cutoff value were scored as positive.

### Repeatability test

In regard to the repeatability evaluation, the DAS-ELISA assay was utilized to detect 10 positive samples and 10 negative samples based on optimal conditions described in the results. The positive samples were artificially contaminated beef, lettuce, and cattle feces with 10^6^ CFU/mL EHEC O157:H7 EDL933 strain culture. The negative samples were pathogen-free beef, lettuce, and cattle feces. Each sample was tested in triplicate in one plate for intra-repeat assay, and results in two plates regarded as inter-repeat assay. The intra- and inter-assay coefficients of variation (% CV) were calculated by the following formula: % CV = standard deviations (SD)/ mean OD_450_ of samples ×100%.

### EHEC O157:H7 measurement in clinical samples

#### Sample preparation

To all samples was added PBS containing 10% glycerol after filtering the samples through a 0.45-μM membrane in PBS. Samples can be frozen, refrigerated, or assayed immediately. For this study, 198 cattle fecal, 48 raw milk, 73 drinking water contaminated with cattle feces, 60 vegetable, 62 beef, and 57 fish samples were collected and assayed with DAS-ELISA and duplex-PCR. Sensitivity, specificity, and accuracy were calculated using = true positive/(true positive + false negative) × 100%; specificity = true negative/(true negative + false positive) × 100%; accuracy = (true positive +true negative)/ (true positive+ false positive + true negative + false negative) × 100%.

#### Sample measurement

Each sample was enriched with 50 mL mEC broth containing novobiocin and tellurite at 41°C for 12 h. Finally, 100 μL cultures were assayed with the DAS-ELISA according to steps described previously.

#### Statistical analysis

Data for OD_450_ values from different samples were expressed as the mean ± standard deviation (SD) using simple statistics in Excel. The data obtained from specificity test of DAS-ELISA were compared in SPSS version 19 using a *t*- test. *P* > 0.05 was considered to be no significance of difference.

## Results

### Protein expression and purification

Recombinant plasmid pCold I-C1 *eae* and pCold I-C2 *eae* were sequenced using Genscript Biotechnology Co. Ltd. (Nanjing, China), sequencing data indicate that C1 *eae* and C2 *eae* have 100% identity to reference sequences of *eae* γ (GenBank Z11541.1). Recombinant bacteria BL21/pCold I-C1*eae* and BL21/pCold I-C1*eae* were induced by IPTG. SDS-PAGE showed that C1-intimin γ1 (32.03 kDa) and C2-intimin γ1 (13.60 kDa) were successfully expressed with 20 and 35% proportion to whole bacterial protein in contrast to naïve bacteria.

### Development and characterization of mAbs against C2-Intimin γ1

Seven hybridomas developed from fusing spleen lymphocytes with SP2/0 cells were reactive to recombinant C2-intimin γ1 of the EHEC O157:H7 EDL933 according to initial screenings, but five were positive after indirect-ELISA retesting following previously described procedures [19].

**Fig 1:**
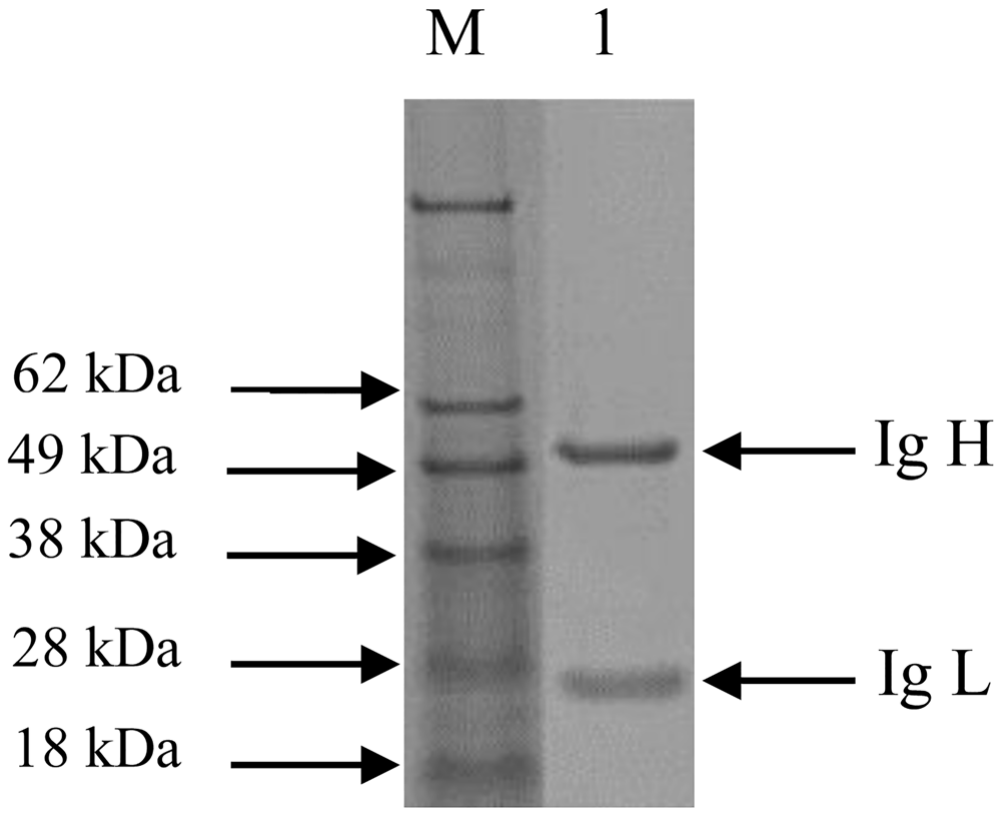
SDS-PAGE of purified ascites. Mouse ascites were purified using Protein A IgG Purification Kit (ThermoFisher). Bands of 55 kDa and 25 kDa were identified as heavy and light chains Lane 1, purified mouse IgG; M: protein molecular weight marker.

All five hybridomas were selected for sub-cloning and all survived the cloning procedure. Ascites were prepared with 2B10, 4D7, and 4C4 lines, and preliminary testing confirmed that mAb 2B10 and 4C4 could react with only EHEC O157:H7 and did not cross react with other *E*.*coli* serotypes and pathogens listed in Table 1. The titers of mAbs were tested by indirect ELISA as abovementioned. The mAb 2B10 and 4C4 had different level antibody titers, they were 2.5 × 10^5^ and 5.12 × 10^5^, respectively. The purified mAb 4C4 from mouse ascites was used for DAS-ELISA development. After purification of mouse ascites, two bands corresponding to 55 kDa and 25 kDa were identified with SDS-PAGE gel (Fig 1) and were heavy and light chains of IgG recognized by HRP-goat anti-mouse IgG (H/L chains) antibodies.

### Development and characterization of pAb against C1-Intimin γ1

**Fig 2:**
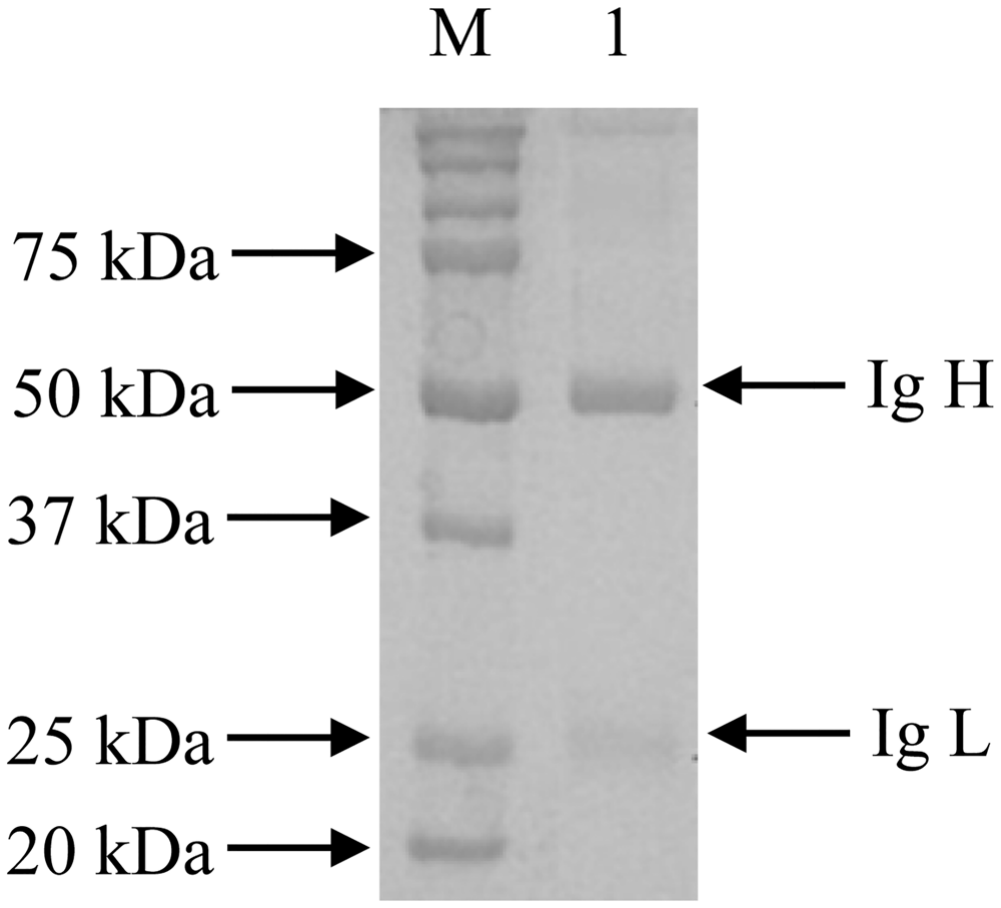
SDS-PAGE analysis of purified rabbit polyclonal antibody. The Thermo Scientific Protein A IgG Purification Kit was used to purify rabbit sera. Two bands of 55 kDa and 25 kDa were confirmed with Western blot to be heavy and light chains. Lane 1, purified rabbit IgG; M: protein molecular weight marker.

Anti-C1-intimin γ was successfully prepared from rabbits 141 and 142, and titers were measured using indirect ELISA and purified with a Protein A IgG Purification Kit. Rabbit serum titers were respective 1.02 × 10^6^ and 3.3 × 10^6^ and after purification, 55 kDa and 25 kDa bands were observed with SDS-PAGE (Fig 2).

### Development of DAS-ELISA

As the general criteria for the development of the sandwich ELISA, antibody concentrations were chosen such that the sensitivity of ELISA was sufficient while nonspecific binding (background) was still low. This led to the following optimal conditions based on ratios from positive to negative wells and individual values [22]. Here, when OD_450_ values of positive sample were ≥1 and negative values were ≤0.2, they had the greatest pos/neg ratios (Table 2), so we selected the corresponding concentration as an optimal antibody concentration. The optimized DAS-ELISA protocol as following: The 96-well plates were coated with 2.5 μg/mL of purified rabbit pAb (capture antibody) diluted in sodium carbonate buffer (pH 9.6) at 4°C overnight. The most suitable blocking buffer was 5% skim milk. The optimal detection antibody concentration was 5.0 μg/mL of purified mAb diluted in PBST. All incubations were carried out at 37°C for 1 h (except incubation for color development with substrate). All washing steps were 5 min for 3 times at room temperature.

**Table 2:** Checkerboard test for optimal mAb and pAb concentrations.

			pAb (μg/mL)					
mAb (μg/mL)		sera	20	10	5	2.5	1.25	0.625
			A	B	C	D	E	F
20	**A**	+	1.52±0.01	1.46±0.02	1.19±0.04	1.07±0.07	0.88±0.02	0.66±0.01
		-	0.44±0.02	0.29±0.00	0.21±0.01	0.28±0.06	0.19±0.01	0.13±0.00
10	**B**	+	1.47±0.01	1.35±0.04	1.23±0.03	1.10±0.02	0.93±0.03	0.69±0.08
		-	0.27±0.02	0.32±0.01	0.25±0.07	0.16±0.02	0.14±0.02	0.11±0.01
5	**C**	+	1.26±0.07	1.18±0.09	1.15±0.05	**1.10±0.01**	0.78±0.02	0.51±0.03
		-	0.22±0.03	0.17±0.02	0.16±0.00	**0.15±0.00**	0.11±0.01	0.09±0.00
2.5	**D**	+	0.95±0.01	0.80±0.04	0.71±0.01	0.56±0.00	0.40±0.01	0.27±0.01
		-	0.25±0.02	0.19±0.01	0.12±0.00	0.12±0.01	0.08±0.01	0.07±0.01
1.25	**E**	+	0.76±0.05	0.57±0.04	0.48±0.01	0.42±0.00	0.29±0.02	0.18±0.00
		-	0.18±0.02	0.23±0.00	0.145±0.04	0.10±0.00	0.08±0.01	0.08±0.01
0.625	**F**	+	0.59±0.00	0.47±0.06	0.40±0.00	0.27±0.08	0.19±0.05	0.18±0.01
		-	0.17±0.01	0.19±0.00	0.09±0.00	0.07±0.00	0.07±0.00	0.06±0.03

Bold numbers show values for optimal DAS-ELISA. Positive value is 1.10 and negative value is 0.15 (ratio 7.35). All experiments were conducted in triplicate. Data are expressed as means ± SD.

### Sensitivity and specificity of DAS-ELISA

**Fig 3:**
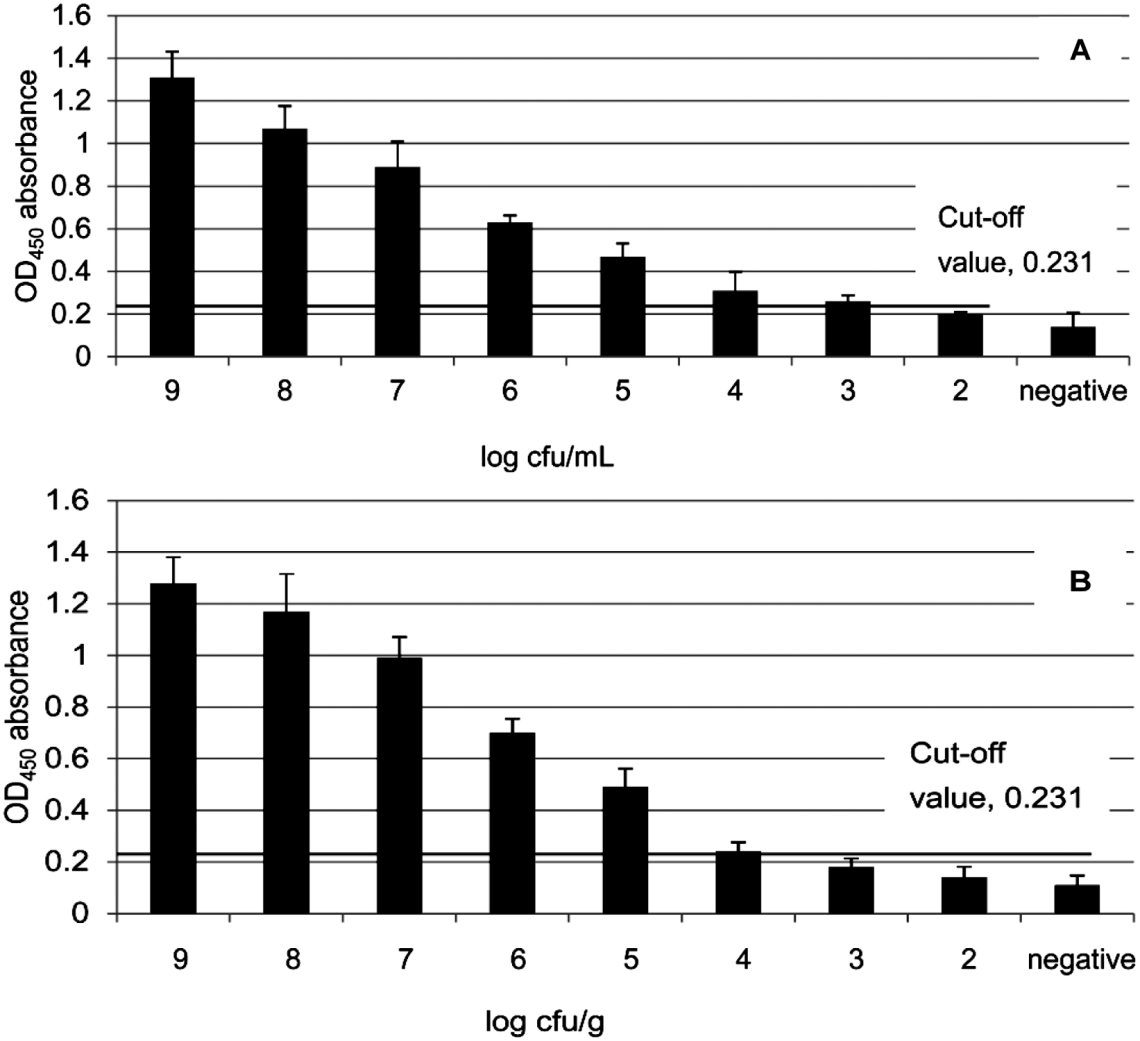
Sensitivity of sandwich ELISA for measuring EHEC O157:H7. DAS-ELISA had a detection limit of 1 × 10^3^ CFU/mL (OD_450_ = 0.26, column 3) for EHEC O157:H7 pure culture (A) and 1 × 10^4^ CFU/g (OD450 = 0.24, column 4) for artificial samples before enrichment (B). All experiments were conducted in triplicate. Data are expressed as average ± SD.

Some variability was observed regarding sensitivity tests carried out with different samples. For EHEC O157:H7 pure cultures, serial dilutions of 10^9^ CFU/mL to 10^2^ CFU/mL were used for the sandwich ELISA. For direct measurement of EHEC O157:H7 pure cultures, the limit was 1 × 10^3^ CFU/mL, corresponding to an OD_450_ of 0.26 (column 3 Fig 3A). For directly measuring artificially contaminated samples, 10^9^ to 10^2^ CFU/g was used for the ELISA, and this offered a sensitivity of 1 × 10^4^ CFU/g which corresponded to an OD_450_ of 0.24 (column 4 Fig 3B), and a sensitivity of 1 × 10^2^ CFU/g for measuring enriched samples (data not shown). For duplex-PCR, the detection limit for enriched artificially contaminated samples was 10 CFU/g, 10 times higher than that of the DAS-ELISA.

**Fig 4:**
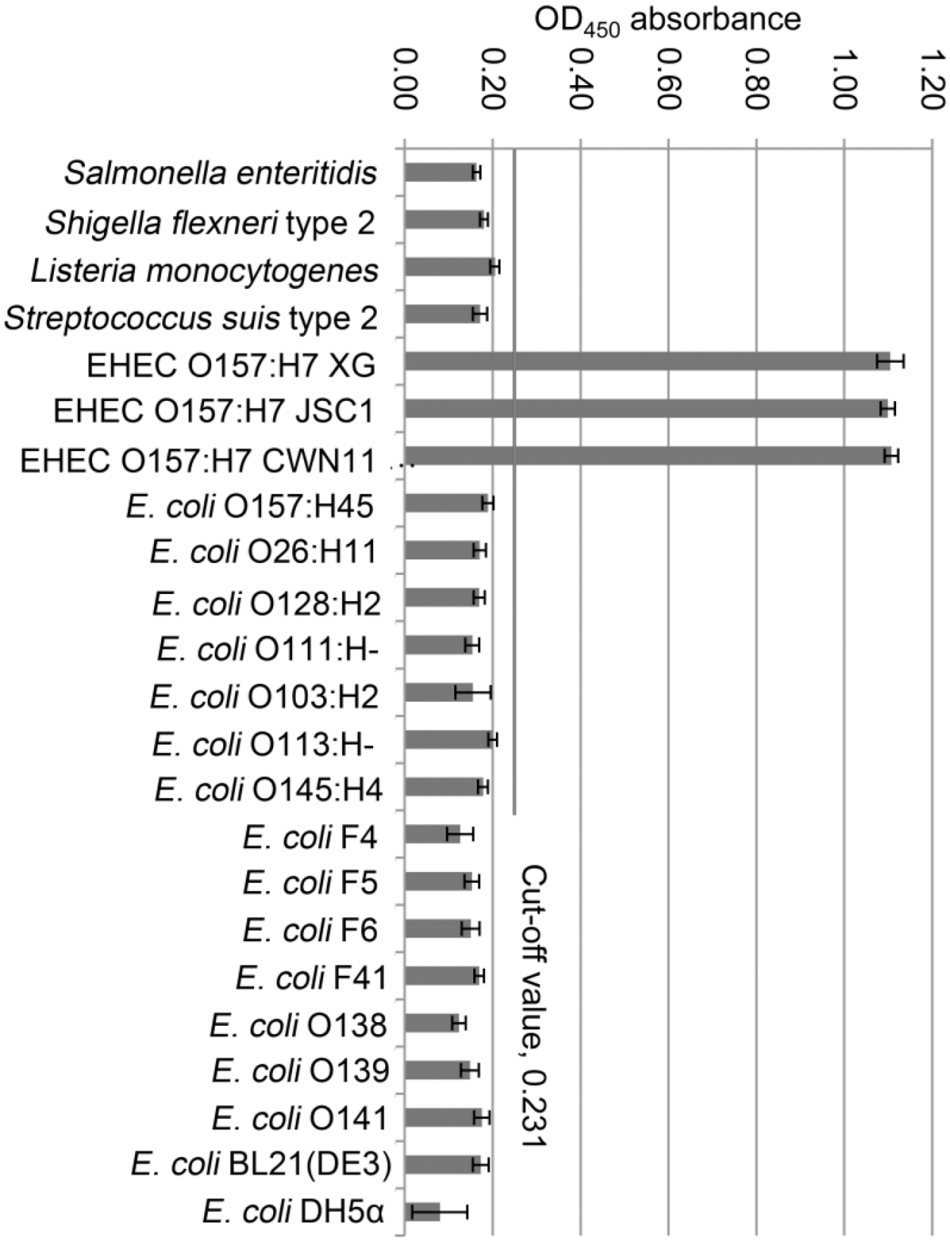
Cross-reactivity test of sandwich ELISA. Four other genera of bacterial pathogens and 19 serotypes of *E*.*coli* were evaluated with DAS-ELISA, and only EHEC O157:H7 had a positive value based on a threshold of OD_450_. When OD_450_ > 0.231, samples were positive, when OD450 ≤0.231, values were negative and not cross-reactive with EHEC O157:H7. All experiments were conducted in triplicate. Data are expressed as means ± SD.

The specificity of DAS-ELISA was evaluated using various foodborne pathogens (See Table 1). EHEC O157:H7 was the only positive strain (OD_450_ = 0.67). The sandwich ELISA was effective for confirming EHEC O157:H7 and was not cross-reactive with other bacterial genera or *E*.*coli* serotypes (Fig 4). In contrast, duplex-PCR was able to amplify bands from genome extracted from *E*.*coli* O157:H45, cross-reaction with non-O157:H7 strains.

### Negative/positive threshold

The threshold was calculated from OD values from fifty negative samples for EHEC O157:H7 as 0.231. When OD_450_ nm values were equal to or greater than 0.231, the sample was considered positive, and if not, the sample was considered negative.

### Stability of DAS-ELISA

The mean %CV of the intra- and inter-batch duplicability tests was 3.31% and 8.2%, respectively, and this was adequate for EHEC O157:H7 detection.

### Detection of EHEC O157:H7 in field samples

A total of 498 field samples (Table 3) from cattle farms and local markets were screened for EHEC O157:H7 using DAS-ELISA and duplex-PCR. With the DAS-ELISA, 67 of 498 field samples were positive, whereas 71 samples were positive according to duplex-PCR. Among 498 samples, 65 were positive with DAS-ELISA and duplex-PCR, and 403 were negative for both tests. DAS-ELISA had 94.38% specificity (403/427) and 91.54% sensitivity (65/71) relative to duplex-PCR. Data from DAS-ELISA and duplex-PCR indicate an accuracy of 93.98% (65 + 403)/(65 + 6 + 403 + 24) between both methods.

**Table 3:** Comparison of DAS-ELISA with duplex-PCR for field samples detection.

	DAS-ELISA			duplex-PCR		Both	
Samples	total	+	-	+	-	+	-
cow swab	198	33	156	35	154	32	147
raw milk	48	2	46	3	45	2	45
water contaminated with cow feces	73	12	61	12	61	11	61
vegetable	60	7	53	7	53	7	53
beef	62	3	59	3	59	3	55
fish	57	10	47	11	46	10	42
total	498	67	431	71	427	65	403

## Discussion

Intimin, an outer membrane protein (OMP) encoded by the locus of an enterocyte effacement (LEE) island, is responsible for the tight association of the pathogen with the host cell. Intimin has been successfully used in immunological-based assay development [23–26]. The variable 3’-region of Intimin has been used to confirm *eae* types and subtypes [3–5]. EHEC O157:H7 is an Intimin γ1 subtype, and the C terminus of Intimin γ1 is conserved in serotype O157:H7, so it is an ideal target for producing antibodies specifically against EHEC O157:H7. Previously, we successfully used the C terminus of Intimin γ1 to construct a multivalent immunogen of H7-HCP-Tir-Intimin, which significantly reduced colonization and shedding of EHEC O157:H7 in goats [27]. Thus, the C terminal region of Intimin γ1 is a specific antigen to develop antibody for EHEC O157:H7 detection.

The DAS-ELISA described here was developed with the polyclonal antibody against C1-Intimin γ1 as the capture antibody and the monoclonal antibody 4C4 against C2-Intimin γ1 as the detection antibody. The procedure for detecting EHEC O157:H7 is rapid, sensitive and specific. The sensitivity of the DAS-ELISA was 100 times higher than that of the conventional double antibody sandwich ELISA based on long-chain lipopolysaccharide from EHEC O157:H7, the detection limit was 1×10^5^ CFU/ml in pure culture [16]. The DAS-ELISA in this study could detect as low as 10^3^ CFU/ml in pure culture, the same as that of duplex-PCR [17, 20]. The specificity of DAS-ELISA was characterized by every non-O157:H7 strain and other genre bacteria used in this study with negative response. However, in the similar ELISA method developed by Kerr’s group, the cross reaction appeared [16], the ELISA was positive with other bacteria, including *Salmonella urbana* strain S127490 and *Vibrio cholera* O1 Inaba strain SC1074.

Sandwich ELISA is widely used to detect the presence of substances, including bacteria, viruses, et al. However, the detection limit of sandwich ELISA to EHEC O157:H7 is only 10^5^ to 10^7^ CFU/ ml [28], which is inadequate when the infectious dose is very lower in samples. In our study, the sensitivity of the DAS-ELISA is higher than similar methods [16], which is relatively important to detect low loading EHEC O157:H7 in samples. The detection limit of the DAS-ELISA described here was 1 × 10^4^ CFU/g in contaminated samples and 1 × 10^2^ CFU/g for measuring enriched contaminated samples. The mAb 4C4 was specific to EHEC O157:H7 and did not react with other common foodborne bacteria and other non-O157:H7 *E*.*coli* strains except EPEC O55:H7 (data not shown), which was the only detected limitation of the DAS-ELISA. The EPEC O55:H7 serotype was a recent precursor to the virulent EHEC O157:H7, and both contained LEE islands which produced Intimin γ1 [29], To our knowledge, current optimal immunology-based assays cannot differentiate them.

To increase specificity, DAS-ELISAs are typically developed with a mAb as a capture antibody and a pAb as a detection antibody. Here, when the mAb was used as a capture antibody the negative control OD_450_ value was higher than expected. To resolve this issue, the pAb was used as a capture antibody, resulting in OD_450_ values for positive samples >1 and those of negative samples were <0.2, as expected (Table 2). When developing DAS-ELISA, selection of capture and detection antibodies depended on target antigens. Previously, a sandwich ELISA for EHEC O157:H7 was developed to detect animal and human samples based on two mAbs against the long-chain lipopolysaccharide from EHEC O157:H7, and this was cross-reactive against bacteria except for EHEC O157 strains [16]. To measure O157 and non-O157 STECs, a sandwich ELISA assay was developed with the same pAb against Stx2 for capture and detection, and reliable sensitivity and specificity was obtained [30]. For *Listeria spp*., an important foodborne bacterium, a sandwich ELISA was developed by comparing selection antibody influences on detection limits. Finally, a mAb was used as capture antibody, and a pAb was chosen for the detection antibody; sensitivity was better than that for a pair of mAbs [31]. Thus, both the antibody against the specific peptide and their matches are two critical factors for developing a reliable and applicable immunology-based assay.

In spite of specific antibodies being necessary for developing DAS-ELISA, original sample storage and treatments contribute to sensitivity and specificity. Here, all samples were saved in 10% glycerol buffer to reduce interference of EHEC O157:H7. Glycerol at 5% and more can inhibit bacterial growth [32], which kept samples safe and favored easier isolation of target pathogens. Also, when many samples were collected from fields, glycerol buffer protected bacteria from freezing. EHEC O157:H7 is mainly colonized in the lower intestine of animals, and their excreta contaminate water, food, and the environment [2]; so, infected and contaminated samples must be identified. Selective enrichment is a major tool for foodborne bacterial detection, and EHEC O157:H7 is an important foodborne bacterium for which sample enrichment procedures have been reported.

Using published methods [16, 23, 30, 33] with some modifications, we diluted original solid samples to 1:1 (v/v) mixtures, centrifuged them at 500 rpm to remove impurities, and then centrifuged supernatant at 16,000 rpm to create a bacterial pellet for selective enrichment. For liquids, filtered membranes were selectively enriched and optimized enrichment procedures increased the sensitivity of the DAS-ELISA. Thus, we developed a simple and unique immunoassay for measuring EHEC O157:H7 using novel mAb and pAb, and the few limitations to the assay will require additional study in the future.
